# A Wireless Bi-Directional Brain–Computer Interface Supporting Both Bluetooth and Wi-Fi Transmission

**DOI:** 10.3390/mi15111283

**Published:** 2024-10-22

**Authors:** Wei Ji, Haoyang Su, Shuang Jin, Ye Tian, Gen Li, Yingkang Yang, Jiazhi Li, Zhitao Zhou, Xiaoling Wei, Tiger H. Tao, Lunming Qin, Yifei Ye, Liuyang Sun

**Affiliations:** 1 College of Electronics and Information Engineering, Shanghai University of Electric Power, Shanghai 201306, China; jiw_5341@163.com (W.J.); lunming.qin@shiep.edu.cn (L.Q.); 2 2020 X-Lab, Shanghai Institute of Microsystem and Information Technology, Chinese Academy of Sciences, Shanghai 200050, China; suhaoyang23@mails.ucas.ac.cn (H.S.); jin6659@mail.sim.ac.cn (S.J.); tiany_field@mail.sim.ac.cn (Y.T.); li1901@foxmail.com (G.L.); ykyang@mail.sim.ac.cn (Y.Y.); jiazhi.li@mail.sim.ac.cn (J.L.); tiger@mail.sim.ac.cn (T.H.T.); 3 School of Graduate Study, University of Chinese Academy of Sciences, Beijing 100049, China; ztzhou@mail.sim.ac.cn (Z.Z.); xlwei-jerry@mail.sim.ac.cn (X.W.); 4 State Key Laboratory of Transducer Technology, Shanghai Institute of Microsystem and Information Technology, Chinese Academy of Sciences, Shanghai 200050, China; 5 Center of Materials Science and Optoelectronics Engineering, University of Chinese Academy of Sciences, Beijing 100049, China; 6 School of Physical Science and Technology, ShanghaiTech University, Shanghai 201210, China; 7 Center for Excellence in Brain Science and Intelligence Technology, Chinese Academy of Sciences, Shanghai 200031, China; 8 Neuroxess Co., Ltd. (Jiangxi), Nanchang 330029, China; 9 Guangdong Institute of Intelligence Science and Technology, Hengqin, Zhuhai 519031, China; 10 Tianqiao and Chrissy Chen Institute for Translational Research, Shanghai 200020, China

**Keywords:** brain–computer interface, wireless transmission, Bluetooth, Wi-Fi

## Abstract

Wireless neural signal transmission is essential for both neuroscience research and neural disorder therapies. However, conventional wireless systems are often constrained by low sampling rates, limited channel counts, and their support of only a single transmission mode. Here, we developed a wireless bi-directional brain–computer interface system featuring dual transmission modes. This system supports both low-power Bluetooth transmission and high-sampling-rate Wi-Fi transmission, providing flexibility for various application scenarios. The Bluetooth mode, with a maximum sampling rate of 14.4 kS/s, is well suited for detecting low-frequency signals, as demonstrated by both in vitro recordings of signals from 10 to 50 Hz and in vivo recordings of 16-channel local field potentials in mice. More importantly, the Wi-Fi mode, offering a maximum sampling rate of 56.8 kS/s, is optimized for recording high-frequency signals. This capability was validated through in vitro recordings of signals from 500 to 2000 Hz and in vivo recordings of single-neuron spike firings with amplitudes reaching hundreds of microvolts and high signal-to-noise ratios. Additionally, the system incorporates a wireless stimulation function capable of delivering current pulses up to 2.55 mA, with adjustable pulse width and polarity. Overall, this dual-mode system provides an efficient and flexible solution for both neural recording and stimulation applications.

## 1. Introduction

Understanding the mechanisms of information transfer and processing in the nervous system is crucial for both basic neuroscience research and the development of therapies for neurological disorders [1,2,3]. Brain–computer interfaces (BCIs) provide a valuable platform for probing neural dynamics by establishing a direct communication pathway between the brain and external devices, allowing for the recording of neural signals and the delivery of targeted stimulation [4,5,6].

Among the various neural signals, action potentials, millisecond-long spikes in cell membrane voltage, serve as the fundamental units of neural information [7]. Capturing and interpreting these spikes is crucial for applications in neuroprosthetics, such as robotic arm control and brain-to-text communication, to restore lost functions to patients [8,9,10]. In addition to action potentials, local field potentials (LFPs) represent lower-frequency neural signals that reflect both spikes and membrane potential fluctuations within localized neural populations [11]. These signals are also important for tracking neural activity. LFP monitoring in specific brain regions has been utilized for diagnosing neurological diseases, such as Parkinson’s disease and epilepsy, and is often integrated into closed-loop stimulation treatments [12,13,14,15].

An ideal BCI system should be capable of high-throughput neural recording, while also offering customizable stimulation pulses, all within a compact, wireless configuration suitable for freely moving subjects. However, since recording action potentials requires high sampling rates, most BCIs have relied on wired systems to ensure the quality of signal transmission [16,17,18]. Although effective, wired systems pose practical limitations in research and clinical environments, restricting subject mobility, introducing interference, and complicating setups with physical connections.

Wireless BCI systems provide greater flexibility, allowing for natural movement and more convenient experimental setups [19,20,21]. Despite these advantages, balancing the transmission quality and power consumption presents a significant technical challenge [22]. Most wireless systems utilize Bluetooth for data transmission due to its low power consumption, making it suitable for long-term, portable applications. However, Bluetooth’s limited data throughput constrains its sampling rate, which restricts many systems to recording low-frequency LFPs and precludes high-frequency spike recordings [23,24,25,26]. While LFPs have clinical value, particularly in the diagnosis of neurological disorders, the inability of most Bluetooth-based BCIs to record high-frequency spikes limits their application to studying rapid neural dynamics and complex neural circuit activities [27]. Wi-Fi, by contrast, offers a significantly higher transmission bandwidth, supporting the higher sampling rates necessary for recording detailed spike data [28,29]. This makes Wi-Fi an appealing option for applications that require high-resolution neural data. However, its increased power consumption presents challenges, especially for long-term monitoring scenarios. Current wireless BCI systems typically rely on a single transmission mode, such as Bluetooth or Wi-Fi, which limits their flexibility to meet the diverse requirements of both research and clinical applications.

To address these limitations, we developed a wireless bi-directional brain–computer interface with dual transmission modes. This system supports both low-power Bluetooth transmission and high-sampling-rate Wi-Fi transmission, allowing for its flexible adaptation to different applications. The Bluetooth mode, with a maximum sampling rate of 14.4 kS/s, is sufficient for detecting low-frequency local field potentials, while the Wi-Fi mode, offering a maximum sampling rate of 56.8 kS/s, is optimized for recording high-frequency spike firings. The in vitro tests confirmed its efficient signal recording across a frequency range of 10 to 2000 Hz, covering the neural signal frequency bands of interest for both LFPs and spikes. Additionally, the system supports wireless biphasic current pulse output for neural stimulation. The in vivo tests on mice demonstrated that the device can capture both LFPs and action potentials from individual neurons with high signal-to-noise ratios, offering a flexible and efficient solution for neural recording and stimulation applications.

## 2. Materials and Methods

### 2.1. System Design

This wireless BCI system was designed as an effective tool for neural signal recording and neuromodulation, allowing for bi-directional interaction with the subject. The system incorporates both Bluetooth and Wi-Fi transmission modes to support a wide range of applications.

#### 2.1.1. Circuit Design

To achieve the miniaturization and lightweight construction of the wireless device, careful consideration was given to both the components selection and layout, optimizing the onboard component density. The core of the device is a microcontroller unit (MCU) chosen for its strong performance, abundant on-chip resources, and integrated RF capabilities, allowing for future functional expansion, such as adding new features and implementing advanced algorithms. A low-power, highly integrated stimulator/amplifier chip was selected to support multi-channel, high-precision neural signal sampling.

Ensuring the power stability is crucial for maintaining consistent device performance. For this reason, low-power, high-efficiency buck–boost regulators were used to support a continuous high-load operation. To protect the circuitry from static electricity or surge impacts caused by power fluctuations, bi-directional electrostatic discharge (ESD) protection diodes were incorporated to clamp excess voltage and prevent potential damage.

In line with the requirements that the device be miniaturized and lightweight, the battery was optimized for energy density, capacity, and size. The charge and discharge circuits were carefully designed to ensure efficient power management. For the passive components, such as the capacitors, inductors, and resistors, compact surface-mount packages were selected to conserve board space.

To address the bulkiness and operational limitations of conventional mechanical switches, a magnetically controlled sensing switch was implemented. This solution provided a more space-efficient and user-friendly alternative.

The device employs a multi-layer printed circuit board (PCB) design, incorporating a dedicated ground plane to mitigate heat accumulation during high-power operation and improve its resistance to electromagnetic interference. In the areas prone to high currents, additional copper plating was used to improve heat dissipation, and heat dissipation holes were strategically placed around the components that are likely to generate heat.

#### 2.1.2. Firmware Design

The development of firmware was essential for managing the system’s high-speed and real-time control tasks. The following sections outline the key functionalities of the firmware.

(1) System Execution Logic

**Figure 1:**
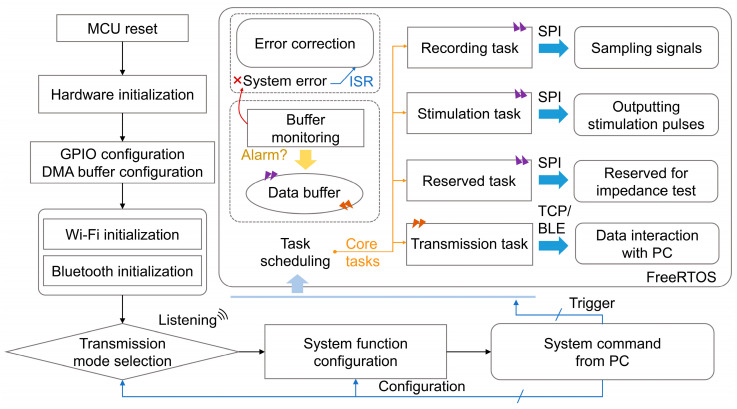
Execution logic of wireless dual-mode BCI system.

The system’s execution logic is illustrated in Figure 1. Upon powering up, the device resets the MCU, triggering the hardware setup and verification processes required for normal operation. This is followed by the configuration of the peripheral registers to enable the desired functionalities. A general-purpose input/output (GPIO) pin allocation is aligned with the PCB’s IO settings. To handle the high-speed sampling data and RF module transmission frequency, two equally sized direct memory access (DMA) buffers are configured in a double-buffer structure, improving the system’s response speed and real-time data bandwidth.

After initialization, the system proceeds to activate either the Wi-Fi or Bluetooth mode. The Wi-Fi initialization consists of four main steps: initializing the non-volatile storage (NVS), setting up the TCP/IP core for data transmission, initializing the Wi-Fi driver, and configuring the Wi-Fi mode, followed by the parameter settings and activation. Similarly, the Bluetooth initialization involves starting the Bluetooth controller; initiating the host and Bluetooth stacks; registering the generic access profile (GAP) and generic attribute profile (GATT) services, along with their callback functions, to handle specific events; configuring the broadcast parameters; and starting the broadcasting to await pairing.

Once the Wi-Fi or Bluetooth setup is complete, the device listens for external connection requests. The host computer selects the desired transmission mode, sends a connection request, and the device responds by suspending the unused mode and releasing its resources. Given the system’s real-time and complex operation, a lightweight real-time operating system (FreeRTOS) is used. The workflow is divided into four core tasks: recording, stimulation, reserved, and transmission tasks, each handling specific system functions. A wireless event group and multiple message queues are created to enable inter-task communication and data exchange. The operating system handles resource scheduling and logic management to ensure an efficient workflow.

Additionally, an error supervision mechanism for the DMA buffer is in place to prevent overwriting errors during recording. If data flow mismatches lead to buffer issues, the system triggers an error-handling routine, which is managed through event-driven callback functions.

(2) Recording Function

The recording process is managed by the recording task. The stimulator/amplifier chip handles the front-end signal acquisition and communicates bi-directionally with the MCU’s SPI controller via SPI communication. The MCU is responsible for sending instructions to the stimulator/amplifier chip, storing data, and wirelessly transmitting the data to the PC.

To enable flexible signal recording, the system supports selective channel configuration. Users can select the recording channels via the graphical user interface (GUI) on the PC. The selected channels are stored as a 16-bit control frame. For instance, if channels 0 and 1 are enabled while the others are disabled, the control frame would be 16’b0000_0000_0000_0011. This control frame is then sent from the PC to the MCU, which automatically configures the selected channels and sends the corresponding command to the stimulator/amplifier chip for signal acquisition.

**Figure 2:**
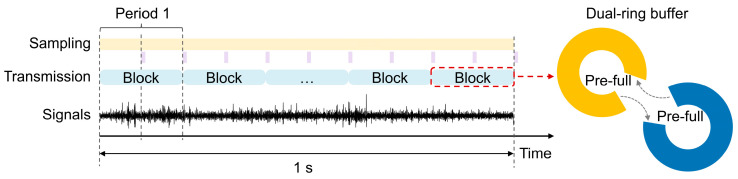
Dual-ring buffer mechanism for signal recording.

Regarding data storage, traditional CPU-based methods can increase system overhead. To improve communication efficiency and system stability, we implemented a dual-ring buffer mechanism (Figure 2), which allows for the data to be directly transferred from the SPI controller to the MCU’s internal random access memory (RAM), reducing the CPU’s load. The specific process is as follows: two equal-sized DMA buffers are pre-allocated in the RAM. Once one of the buffers reaches the set threshold, a signal is triggered to activate the transmission task. The MCU then initiates the internal radio module and transmits the collected data in blocks to the host computer in real-time using the pre-selected communication method (Wi-Fi or Bluetooth).

During the design phase, several parameters related to the sampling process were tested, including the SPI controller clock frequency, transaction queue length, control mode, DMA buffer size, and Wi-Fi/Bluetooth protocol stack configurations. These tests helped to find an optimal balance between data flow rates, SPI controller efficiency, and MCU performance under varying network conditions. This balance minimizes wireless transmission latency, allowing for a peak downlink rate of up to 1.9 Mbps. In the rare event of severe communication blockages in challenging environments, an error supervision mechanism ensures the system resumes normal operation within 1–2 milliseconds.

(3) Stimulation Function

**Figure 3:**
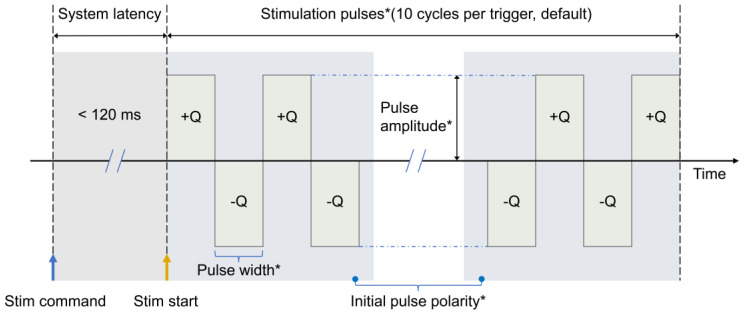
Schematic of stimulation patterns.

The device currently supports two modes of biphasic current pulse stimulation, distinguished by the initial pulse polarity: anodic leading or cathodic leading. Figure 3 illustrates the schematic of the stimulation patterns, where “*” indicates the adjustable parameters. After configuring the stimulation parameters (e.g., pulse width, pulse amplitude) via the host computer, a stimulation sequence is initiated. The delay from the signal trigger to the actual stimulation current generation is approximately 120 ms, with each stimulation comprising 10 consecutive pulses.

**Figure 4:**
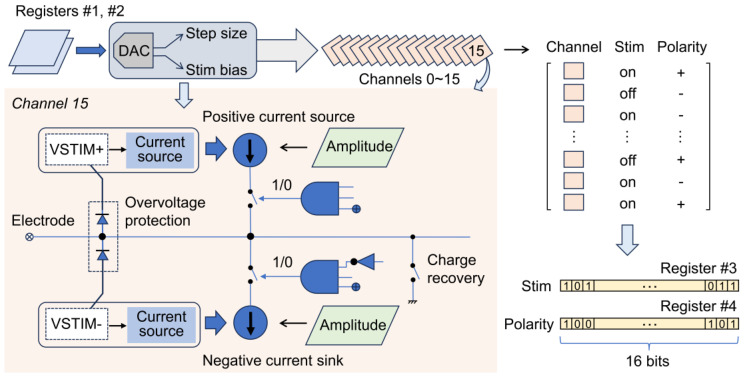
Local circuit of channel stimulator and its control flow.

To achieve these functions, the mainboard control unit configures the stimulation-related digital registers of the stimulator/amplifier chip using a 32-bit SPI control frame. Figure 4 shows the local circuit of the channel stimulator and its control flow. Before stimulation, registers #1 and #2 are configured to adjust the global step size and bias parameters of the 8-bit current output digital-to-analog converter (DAC). The step size affects the current fine-tuning interval and the upper limit for all channels, while the bias optimizes the compliance range of the current driver under the current step size. The output amplitude of each channel can be configured through a specific register, which is set to the maximum value under the current DAC step size in this device.

The polarity selection and stimulation initiation of the different channels are controlled by two separate 16-bit registers (#3 and #4). Each bit of these registers corresponds to a specific channel (0–15). By repeatedly adjusting these registers, the stimulation current waveform can be modified. Before the device is activated, the mainboard control unit initializes the stimulator/amplifier chip and configures the global parameters and amplitude for stimulation, waiting for the configuration and enabling instructions from the host computer. The host computer packages the interface parameters into a one-byte command and sends it to the device, triggering the stimulation function via a button (as shown in Table 1). Upon receiving the configuration parameters, the mainboard control unit decodes them and configures the general-purpose timer (e.g., clock frequency, auto-reload threshold). The timer threshold triggers an interrupt callback function, setting an event flag in the operating system to activate the stimulation task. This task periodically refreshes two specific registers of the stimulator/amplifier chip to control the channel polarity and switching, completing a full cycle of 10 pulses.

(4) Graphical User Interface

A graphical user interface (GUI) was developed using the Qt 5.14.2 framework and C++ programming language to control and monitor the real-time streaming data received by the personal computer (PC). Before initiating the functions, users can customize the system’s operating mode and parameters based on the specific requirements or environment, such as selecting between Wi-Fi and Bluetooth modes or configuring the sampling and stimulation parameters. The received data are stored in a binary format, which can later be processed and analyzed using MATLAB R2023b.

### 2.2. In Vitro Recording and Stimulation

To validate the device’s sampling and transmission capabilities in dual-mode operation, we conducted in vitro tests for both signal recording and stimulation.

For the recording function, since neural signals span multiple frequency bands, a signal generator was used to output sine waves of various frequencies (10–2000 Hz) to test the device’s acquisition capabilities. The testing setup consisted of a wireless BCI device, a microelectrode array (KD-MWA-32, KedouBC, Suzhou, China), a signal generator, a phosphate-buffered saline (PBS) solution, and a PC. Both the electrodes and the signal generator output probe were immersed in the PBS solution, with their reference (ref) terminals connected to a common ground. The signals were then transmitted wirelessly (via Wi-Fi or Bluetooth) to the PC for recording. To evaluate the accuracy, the recorded signals were compared with standard signals at the same sampling rate using a Pearson correlation analysis. The Pearson correlation coefficient was calculated, where a value closer to 1 indicated higher signal similarity and minimal transmission distortion.

For the stimulation function, we verified the device’s ability to generate effective current pulses by measuring the voltage changes across a 1 kΩ resistor. The testing setup included a 1 kΩ resistor, an oscilloscope (DSOX1202G, Keysight Technologies, CA, USA), a microelectrode array (KD-MWA-32, KedouBC, Suzhou, China), a conductive silver adhesive, and a glass substrate. In the experiment, the electrode array was connected in series with the 1 kΩ resistor using the conductive silver adhesive, and the electrode’s reference terminal was linked to one end of the resistor to form a closed circuit. The oscilloscope was connected in parallel with the resistor to record the voltage changes across it. The device’s ability to output current pulses of varying amplitudes (0.51 mA, 2.55 mA), pulse widths (1 ms, 2 ms), and initial polarities (cathodic leading or anodic leading) was validated. The voltage across the resistor was recorded through wireless transmission.

### 2.3. In Vivo Recording

All the animal experiments were approved by the Ethics Committee for Animal Management of the Shanghai Laboratory Animal Research Center (approval number: PA202300702). Eight-week-old male C57BL/6J mice were used in this study, with a microelectrode array (KD-MWA-32, KedouBC, Suzhou, China) implanted into their secondary motor cortex (M2: anteroposterior (AP) + 1.5 mm, mediolateral (ML) ± 0.75 mm, dorsoventral (DV) −1 mm). This brain region is associated with mouse motor functions, featuring highly active neurons, making it suitable for observing neural signal changes [30,31]. Neural signal recording was conducted on the 8th day post-implantation. During the experiment, all-channel signals were recorded using the Bluetooth mode to capture the multi-channel local field potentials. Additionally, two channels were selected for high-sampling-rate recording via Wi-Fi to obtain single-neuron spike firings.

## 3. Results

### 3.1. System Architecture

**Figure 5:**
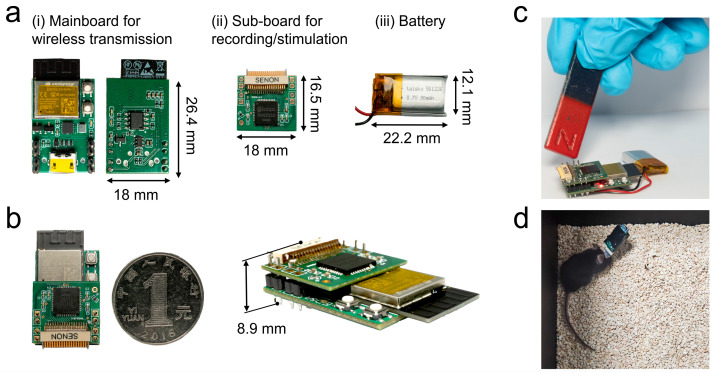
Wireless dual-mode BCI system architecture: (**a**) hardware components, including mainboard, sub-board, and battery; (**b**) stacked connection between mainboard and sub-board; (**c**) magnetic sensing switch for system’s wireless power control; (**d**) mouse equipped with wireless BCI.

The system adopts a stacked architecture, consisting of a mainboard, sub-board, and battery module (Figure 5a). The mainboard (18 mm × 26.4 mm) serves as the core control unit, managing the various system functions, driving the data flow, and controlling the sub-board. The sub-board (18 mm × 16.5 mm) functions as the recording/stimulation unit, directly connected to the front-end electrodes via Omnetics connectors, facilitating neural signal collection and stimulation of the implanted brain region. Both the mainboard and sub-board are built on Flame Retardant 4 (FR4) substrates, with thicknesses of 1 mm and 0.8 mm, respectively. The power module (22.2 mm × 12.1 mm) is a rechargeable lithium battery (taisko 501220) with a nominal capacity of 90 mAh. In terms of battery life, tests revealed that the system can operate for 31 to 34 min in Wi-Fi transmission mode and 51 to 55 min in Bluetooth mode. Given that typical mouse experiments last approximately 10 to 20 min, both transmission modes provide adequate power for standard experimental durations.

The mainboard and sub-board are connected through an eight-pin back-to-back header (Figure 5b), while the battery is connected to the reserved pads on the mainboard via two cables. The entire assembled system weighs 6.2 g (including the 2 g battery) and has overall dimensions of 18 × 26.4 × 15 mm, meeting the requirements for routine experiments with mice (Figure 5d).

Notably, unlike traditional devices, this system uses a magnetic switch instead of a physical switch to enhance experimental convenience. Physical switches are often bulky and can be inconvenient to frequently toggle when on a subject’s head. The magnetic switch (Figure 5c) consists of a bipolar Hall effect switch coupled with a P-type metal–oxide–semiconductor (PMOS) transistor that supports a high current. During practical testing, the magnetic switch demonstrated a fast response time of 30 ms and a lifespan of over tens of thousands of cycles, greatly improving the reliability of the system’s power control.

**Figure 6:**
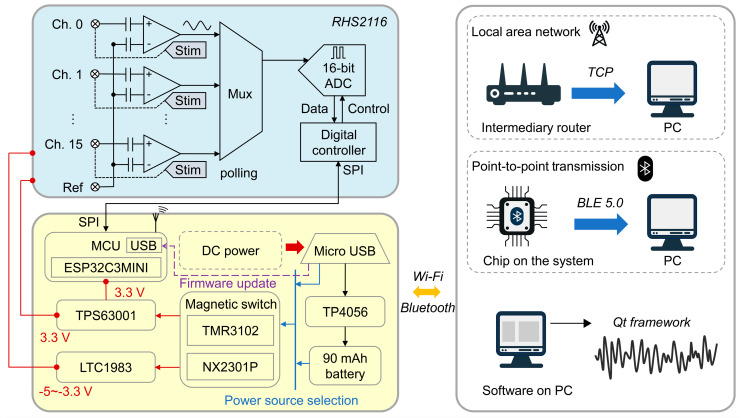
Functional diagram of wireless dual-mode BCI system, illustrating internal data/power connections and wireless transmission to PC.

Figure 6 presents a functional diagram of the wireless dual-mode BCI system. In detail, the system’s mainboard is equipped with a low-power, highly integrated MCU chip (ESP32C3, Espressif Systems, Shanghai, China). The MCU supports 2.4 GHz Wi-Fi and Bluetooth Low Energy (BLE) for wireless communication, and includes a CPU, high-speed SPI controller, GPIO, DMA, and two independent general-purpose timers to facilitate dual-mode sampling and transmission.

The sub-board integrates a stimulator/amplifier chip (RHS2116, Intan Technologies, Los Angeles, CA, USA), which is widely used for electrical signal sampling and supports up to 16 channels for sampling and stimulation. For signal sampling, microvolt-level electrophysiological signals are first amplified by an analog front-end amplifier with high fidelity. These signals are then quantized by a 16-bit high-speed, high-precision analog-to-digital converter (ADC) and acquired based on external control commands. For stimulation, the stimulation current is generated by the internal registers of the RHS2116, configured through a voltage source that passes through a multi-stage current mirror circuit to form a constant current source. This design allows for a customizable pulse width and amplitude of the stimulation waveform, supporting a maximum stimulation current of 2.55 mA and multi-channel concurrency.

The mainboard includes a micro-USB port for lithium battery charging and firmware upgrades. To provide a low-ripple, high-quality power supply to the MCU and RHS2116 while considering component miniaturization and high current output, the device adopts a bi-directional charge pump chip (TPS63001, Texas Instruments, Dallas, TX, USA), which ensures a stable 3.3 V operating voltage. Additionally, an LTC1983 inverter provides a negative voltage for the RHS2116, supplying up to 100 mA for neural stimulation. The charging module uses a TP4056 charging management chip, which features multiple charging and throttling modes tailored to different battery conditions, maximizing the battery’s limited cycle life and extending its service life.

The BLE and Wi-Fi dual-mode communication expands the device’s application scenarios. In BLE mode, the system’s onboard Bluetooth module enables point-to-point communication with a smartphone app or a PC host, making it convenient for low-power scenarios that do not require high sampling rates, such as recording lower-frequency brain signals like alpha waves (8–12 Hz), beta waves (12–30 Hz), gamma waves (30–100 Hz), and local field potentials (<300 Hz). The Wi-Fi mode is configured as a Station mode, utilizing an external router to establish a local area network. In this setup, the router acts as the transmission medium, with the system device functioning as the server and the host computer as the client for intra-network communication. Based on the 802.11b/g/n standard, this mode can be applied in scenarios that require high sampling rates to capture multi-channel local field potentials or high-frequency spike signals from individual channels.

On the host side, the software was developed using the Qt framework, featuring a GUI that allows the user to select the communication mode, control recording, and stimulation functions, and to visualize the data in real-time, facilitating a personalized human–computer interaction experience.

### 3.2. In Vitro Validation of Dual-Mode Recording

In neural research, capturing low-frequency local field potentials (LFPs) and high-frequency action potentials (spikes) provide critical insights into neural activity and its underlying mechanisms. Thus, the capability to reliably record signals across a broad frequency range serves as a key measure of a system’s effectiveness and reliability.

**Figure 7:**
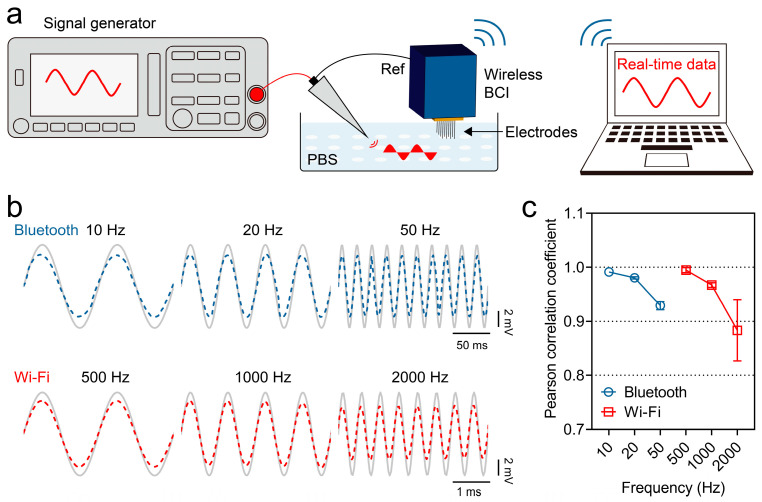
In vitro validation of dual-mode recording. (**a**) Schematic of system setup for in vitro dual-mode recording of standard sine wave signals using wireless BCI. (**b**) Comparison of recorded signals in Bluetooth mode (blue dashed lines) and Wi-Fi mode (red dashed lines) with standard input signals (gray lines). (**c**) Pearson correlation coefficients between recorded signals (Bluetooth and Wi-Fi) and standard signals across different frequencies.

To assess the capacity of our system for capturing signals across a broad frequency range, we conducted in vitro tests using an electrode array to record the standard signals generated by a signal generator. The experiment was set up as shown in Figure 7a. The signal generator outputted standard sine waves at various frequencies with an amplitude of 5 mV. We selected 10, 20, and 50 Hz as the test waveforms in the low-power Bluetooth mode, while we selected 500, 1000, and 2000 Hz for testing in the high-sampling-rate Wi-Fi mode, covering the major frequency bands of neural signals from low to high frequencies.

As shown in Figure 7b, the blue and red lines represent the signals recorded in the Bluetooth and Wi-Fi modes, respectively, while the gray lines represent the standard signals from the source. It can be observed that the recorded signals have a stable amplitude of approximately 4 mV, indicating some attenuation compared to the 5 mV input signal. This attenuation might be due to voltage division during signal transmission. However, despite the amplitude reduction, the waveform shape and frequency of the signals in both the Bluetooth and Wi-Fi modes closely match those of the standard signal, with no significant noise or artifacts.

To quantitatively assess the similarity between the recorded signals and the standard signals, we performed a Pearson correlation analysis to compare their linear correlation (Figure 7c). The Pearson correlation coefficient, which ranges from −1 to 1, measures the linear relationship between two datasets, with values closer to 1 indicating a higher similarity between the waveforms. The results show a slight decrease in the correlation with an increasing frequency, which aligns with the Nyquist sampling theorem, reflecting the inherent limitations of high-frequency signal sampling.

In Bluetooth mode, the correlation coefficients for the low-frequency signals of 10 Hz and 20 Hz exceeded 0.96, and for the 50 Hz signal, the coefficient was 0.929 ± 0.006. In Wi-Fi mode, the reconstruction accuracy of the 1000 Hz signal reached over 0.97, and even at 2000 Hz, the correlation coefficient remained at 0.884 ± 0.046. These results fully demonstrate the high-fidelity signal transmission capability of our device in practical signal testing, proving its ability to reliably capture neural signals across commonly used frequency bands, meeting the requirements for neuroscience research.

### 3.3. In Vitro Validation of Stimulation

To thoroughly validate the device’s ability to generate effective stimulation currents, we designed a comprehensive in vitro test. In this experiment, four channels of the electrode array were selected to output specific current pulses. The effectiveness of the stimulation was verified by monitoring the voltage changes across a 1 kΩ resistor in the test circuit, allowing us to assess the characteristics of the output current pulses. This setup provided a straightforward method to evaluate the performance of the stimulation system.

**Figure 8:**
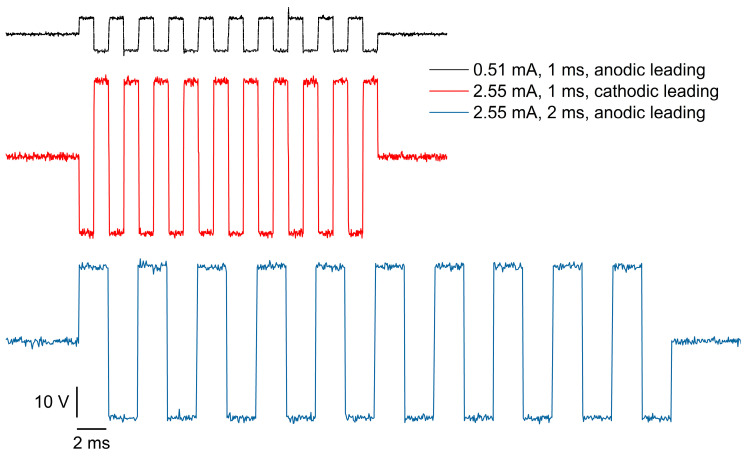
Voltage signals across 1 kΩ resistor under stimulating current pulses with varying parameters: pulse amplitude (0.51 mA, 2.55 mA), pulse width (1 ms, 2 ms), and initial pulse polarity (anodic leading or cathodic leading).

Figure 8 presents the voltage signals captured by the oscilloscope under various stimulation parameters. According to the system’s preset configuration, each stimulation event triggers 10 consecutive current pulses. During the tests, we systematically varied the stimulation parameters, including the pulse amplitude (0.51 mA, 2.55 mA), pulse width (1 ms, 2 ms), and initial pulse polarity (anodic leading or cathodic leading). The measured voltage amplitudes closely matched the input current pulses. Specifically, a 0.51 mA current input induced a voltage of approximately 5 V, while a 2.55 mA current input produced a voltage around 25 V, maintaining a consistent fivefold relationship between the current and the resulting voltage. Additionally, the pulse width precisely aligned with the predefined settings of 1 ms or 2 ms. This consistency in both the amplitude and width across different parameters demonstrates that the device not only provides an accurate and stable current output, but also exhibits versatility in generating various stimulation profiles. These findings confirm the device’s suitability for applications requiring precise neural modulation, where a reliable and controlled stimulation is essential for successful outcomes.

### 3.4. Neural Recording in Mice

**Figure 9:**
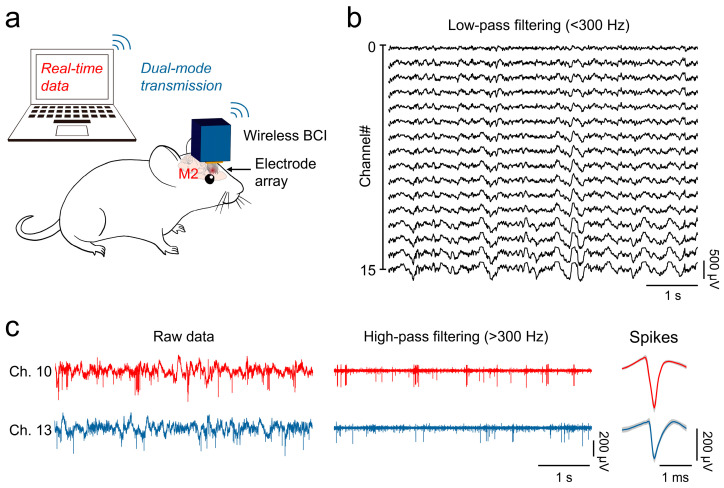
In vivo neural recording in mice. (**a**) Schematic of neural recording setup using a wireless brain–computer interface (BCI) with dual-mode transmission. (**b**) Recorded local field potentials (LFPs) from all 16 channels in Bluetooth mode. (**c**) Neural signals from two channels in Wi-Fi mode, including raw data, high-pass filtered data, and sorted spikes.

After confirming the system’s ability to record broadband signals in vitro, we proceeded with in vivo neural signal detection in mice to validate its capability to capture low-frequency local field potentials (LFPs) and high-frequency action potentials (spikes) (Figure 9a). During the in vivo tests, both the Bluetooth and Wi-Fi modes were employed to capture the neural signals. The Bluetooth mode recorded the signals from all 16 channels at a single-channel sampling rate of 900 Sps, while the Wi-Fi mode focused on 2 specific channels, providing a higher sampling rate of 22 kSps per channel.

Figure 9b shows the neural signals recorded from the mouse’s M2 brain region using the Bluetooth mode. The raw signals, after applying a 300 Hz low-pass filter, display characteristics consistent with those of typical LFPs, indicating the system’s capability to effectively capture low-frequency neural activity.

In the Wi-Fi mode, the system detected numerous distinct spikes. Figure 9c first illustrates the unfiltered raw data from channels 10 and 13, where both low-frequency LFP characteristics and prominent high-frequency spikes can be observed. After applying a 300 Hz high-pass filter, the low-frequency components of the neural signals were removed, isolating the spikes that correspond to the sharp peaks observed in the raw data. Further processing using MountainSort [32] enabled spike sorting, revealing distinct spike waveforms associated with single-neuron firings. The average waveforms are plotted in red and blue, with the shaded gray areas representing the standard deviation of all the detected spikes. The peak-to-peak amplitudes of the recorded spikes are 314.57 μV for channel 10 and 232.03 μV for channel 13, with corresponding signal-to-noise ratios (SNRs) of 30.85 and 13.21, respectively, confirming the high quality of the signal acquisition.

These results demonstrate that the system, in the low-power Bluetooth mode, reliably records the local field potentials, while in the high-sampling-rate Wi-Fi mode, it enables single-neuron-level spike recording. This flexibility in capturing both low- and high-frequency neural signals underscores the system’s effectiveness for comprehensive neural monitoring in vivo.

## 4. Discussion

We developed a wireless bi-directional brain–computer interface (BCI) system that supports both Bluetooth and Wi-Fi transmission, offering versatile solutions for various applications in neuroscience research and clinical studies.

The dual-mode transmission provides flexible options tailored to different research needs. For studies focusing on low-frequency local field potentials (LFPs), such as the diagnosis of neurological disorders like epilepsy and Parkinson’s disease, the Bluetooth mode is advantageous due to its lower sampling rate and reduced power consumption. Our in vitro low-frequency signal recordings and in vivo LFP recordings in mice confirmed the Bluetooth mode’s effectiveness and stability for reliably capturing these signals. In contrast, for neuroscience research that requires decoding based on action potential firings, the Wi-Fi mode, with its higher sampling rate, is more suitable. Our system’s ability to record high-frequency signals was validated both in vitro and in vivo, where the recorded spike firings in the mice demonstrated high signal-to-noise ratios and spike amplitudes of several hundred microvolts.

In addition to signal recording, the system integrates a wireless stimulation function capable of delivering current pulses of up to 2.55 mA, with adjustable pulse width and polarity. The in vitro tests characterized the system’s stimulation capacity, showcasing its potential as a valuable tool for closed-loop BCI research in neural modulation.

A comparative analysis with other recently reported wireless BCI systems reveals that our system performs exceptionally well in terms of its size, weight, number of recording channels, number of stimulation channels, transmission range, and ADC resolution (Table 2). It achieves 16-bit high-precision signal acquisition, supporting a full-channel sampling rate of up to 56.8 kS/s in Wi-Fi mode and 14.4 kS/s in Bluetooth mode. Notably, this system is the only one reported so far that supports both Bluetooth and Wi-Fi communication modes, adding to its versatility.

However, there are areas that require improvement. While the integration of a high-performance MCU with a 160 MHz clock speed allows the device to handle complex tasks and supports its future functional expansion, it also increases its power consumption. One potential approach to address this issue would be to optimize the firmware and refine the circuit design, ensuring improved power efficiency in future iterations. Additionally, minimizing the system’s volume and weight remains a key objective, and there is still room for optimization. For instance, components, such as the two white buttons on the mainboard (Figure 5a), which are primarily used during firmware development, could be removed once the firmware is finalized. The MCU could then be transferred to a smaller board to further reduce the system’s size. Exploring smaller component packages and streamlining the connection between the mainboard and sub-board could also contribute to an overall size reduction. In terms of device packaging, the current simple tape-based solution (Figure 5d) provides basic protection but leaves room for improvement, particularly regarding water resistance and durability. A more advanced approach could involve developing a lightweight 3D-printed shell that could offer better protection and provide a more robust, long-term packaging solution. Lastly, to enhance user experience and flexibility, the development of a mobile or web interface for the real-time control of signal acquisition and device configuration could significantly increase its convenience and usability in experimental settings.

In summary, this wireless bi-directional BCI system is a versatile and robust tool for a wide range of neural applications. With further optimizations, it could hold significant potential for advancing research in brain–computer interfaces.

## Figures and Tables

**Table 1:** System configuration parameters.

Task Configuration ^1^		Function Parameter Configuration ^2^		
Recording	0x01	Sampling rate	b\00	22 kS/s
			b\01	28.4 kS/s
			b\10	900 S/s
			b\11	3.55 kS/s
Stimulation	0x11	Initial pulse polarity	b\00	anodic leading
			b\01	cathodic leading
Reserved	0x10	Pulse amplitude	b\00	0.51 mA
			b\01	2.55 mA
/	/	Pulse width	b\00	1 ms
			b\01	2 ms

^1^ This configuration contains one byte and supports repeated updates while the system is operating. ^2^ This configuration contains one byte and supports a single update after power-up.

**Table 2:** Comparison of this wireless BCI system and other wireless BCI systems.

Publication	[29]	[23]	[24]	[25]	[26]	This Work
Year	2018	2018	2019	2021	2021	2024
Dimensions (mm^3^)	-	44.5 × 16.6 × 9.9	36 × 33 × 15	15 × 15 × 12	43 × 39 × 10	27 × 18 × 15
Total weight	27.73 g	6.87 g	17.95 g	3.9 g	16.8 g	6.2 g
Wireless transmission mode	Wi-Fi	BLE	BLE	BLE	BLE	Wi-Fi/BLE
Support multiple transmission modes	No	No	No	No	No	Yes
Support stimulation [channels]	Yes [8 ch]	No	Yes [8 ch]	No	Yes [-]	Yes [16 ch]
Transmission distance	20 m	5 m	2 m	5 m	-	Wi-Fi 30 m BLE 6 m
Number of channels	8 ch	2 ch	128 ch	1 ch	1 ch	16 ch
ADC resolution	12 bits	16 bits	15 bits	12 bits	10 bits	16 bits
Sampling rate	120 kS/s	40 kS/s	128 kS/s	10 kS/s	1 kS/s	Wi-Fi 56.8 kS/s BLE 14.4 kS/s
Spike detection	Yes	No	No	No	No	Yes
Power consumption	-	45.5 mW	172 mW	-	-	Wi-Fi 590 mW BLE 395 mW

## Data Availability

The original contributions presented in this study are included in the article; further inquiries can be directed to the corresponding authors.
